# Tuned in to communication sounds: Neuronal sensitivity in the túngara frog midbrain to frequency modulated signals

**DOI:** 10.1371/journal.pone.0268383

**Published:** 2022-05-19

**Authors:** Abhilash Ponnath, Michael J. Ryan, Zhide Fang, Hamilton E. Farris

**Affiliations:** 1 Neuroscience Center, School of Medicine, LSUHSC—New Orleans, New Orleans, LA, United States of America; 2 Department of Integrative Biology, University of Texas-Austin, Austin, TX, United States of America; 3 Biostatistics, School of Public Health, LSUHSC—New Orleans, New Orleans, LA, United States of America; 4 Department Cell Biology and Anatomy, School of Medicine, LSUHSC—New Orleans, New Orleans, LA, United States of America; 5 Department of Otolaryngology & Biocommunication, School of Medicine, LSUHSC—New Orleans, New Orleans, LA, United States of America; Claremont Colleges, UNITED STATES

## Abstract

For complex communication signals, it is often difficult to identify the information-bearing elements and their parameters necessary to elicit functional behavior. Consequently, it may be difficult to design stimuli that test how neurons contribute to communicative processing. For túngara frogs (*Physalaemus pustulosus*), however, previous behavioral testing with numerous stimuli showed that a particular frequency modulated (FM) transition in the male call is required to elicit phonotaxis and vocal responses. Modeled on such behavioral experiments, we used awake in vivo recordings of single units in the midbrain to determine if their excitation was biased to behaviorally important FM parameters. Comparisons of stimulus driven action potentials revealed greatest excitation to the behaviorally important FM transition: a downward FM sweep or step that crosses ~600 Hz. Previous studies using long-duration acoustic exposure found immediate early gene expression in many midbrain neurons to be most sensitive to similar FM. However, those data could not determine if FM coding was accomplished by the population and/or individual neurons. Our data suggest both coding schemes could operate, as 1) individual neurons are more sensitive to the behaviorally significant FM transition and 2) when single unit recordings are analytically combined across cells, the combined code can produce high stimulus discrimination (FM vs. noise driven excitation), approaching that found in behavioral discrimination of call vs. noise.

## Introduction

Across animal taxa, receivers are often most sensitive to the stimulus parameters of their species-specific mating signals [1, 2]. This specificity forms the basis of the matched filter hypothesis, which predicts that signaling and sensory processing coevolve, resulting in sensory responses ‘tuned’ to the signal parameters mediating functional behavior [3–5]. For acoustic signals, Suga [6] proposed that information in signals used during recognition and discrimination tasks could be found in three acoustic components or information-bearing elements (IBE): noise bursts, constant frequency, and frequency modulation. Thus, neural filters may be matched to the species-specific acoustic parameters within these elements (i.e., which were termed information-bearing parameters). The mechanisms underlying such filters are known to include specialization in peripheral sensory mechanisms, as well as integrative circuitry in the central nervous system [7–10]. Such neural mechanisms are relatively simple to characterize when signal recognition is based on a single stimulus parameter. For example, for certain insect and frog communication sounds with little spectral complexity (i.e., constant frequency), frequency tuning in the neural periphery may be correlated to the signals’ tonal or limited bandwidth [2, 11–15]. However, communication signals are often more complex and include multiple spectral components that vary with time, as in frequency modulation (FM) [16]. Consequently, characterizing the neural substrate mediating complex species-specific filtering, particularly in single neurons, is often more difficult because: 1, the acoustic parameters of the complex stimulus that are necessary and sufficient to elicit behavior are often difficult to identify; and 2, the integrative circuitry necessary to process the complex stimuli may be anatomically distributed as a hierarchical network or neural population [9, 17]. Using túngara frogs (*Physalaemus pustulosus*), in which these two obstacles have been largely overcome, this project tested whether single neurons in the midbrain, a sensory-motor interface in anurans [18], exhibit greater sensitivity to the particular parameters of male complex calls that are critical to mediating mate recognition behavior and whether this neural sensitivity is sufficient to explain signal discrimination in behavior.

Male túngara frogs produce a complex call consisting of two distinct components. The ‘whine’ is a ~300–400 ms FM sweep in which the dominant frequency changes from ~900–280 Hz. It may be followed by 0–7 harmonic bursts (40–80 ms) called ‘chucks’. Whereas the whine is necessary and sufficient to elicit and direct female phonotaxis, chucks artificially presented alone do not elicit a response. Their addition to the whine increases a whine’s attractiveness and may be used for directional phonotactic decisions when the two components are perceptually grouped [19–22]. The whine’s function is not limited to eliciting female behavior, as it is sufficient to evoke vocal responses from other males [21, 23]. In total, the whine is critical to reproductive behavior, including mediating how other reproductive stimuli (i.e., chucks and visual signals) are processed [24–26]. Previous studies have used stimuli with varied FM structure to determine which parameters of the FM whine elicit responses. For example, whine FM up-sweeps are largely ineffective compared to the natural down-sweep in eliciting responses from males [27–29]. For female phonotaxis, Wilczynski et al. [30] tested individuals given a choice between noise and approximately thirty different whine variants, in which portions of the FM had been removed or altered. Thus, the whine variants were shortened, had gaps, were time reversed (reversed FM), lacked FM, or consisted of FM steps from one tone to another. Their large behavioral dataset revealed that for call recognition there is a 150 ms portion of the whine in which the frequencies of the FM must pass in sequence from a high band (900–560 Hz) to a low band (640–500 Hz) 50 ms later. Thus, their behavioral data generated a model in which túngara frogs are using FM as their IBE and this particular FM transition is considered the information-bearing parameter [6, 9, 31]. That is, whine recognition results from the sequential integration of two parameters: energy from first the high and then the low frequency bands [30].

Where in the auditory system might such FM processing occur? FM sensitivity in single neurons is known to be mediated by several mechanisms including duration tuning, asymmetric facilitation, and delayed lateral inhibition [32–35], strongly implicating convergent circuitry from multiple peripheral frequency channels. At least one early processing stage with such circuitry and evidence for specific FM sensitivity is the auditory midbrain [36, 37]: the torus semicircularis (TS) or its mammalian homologue the inferior colliculus (IC) [38–40]. For example, in bats and rodents the IC contains neurons sensitive to FM stimuli, including specializations for the FM bandwidth, rate, and sweep direction of functionally relevant sounds [41–45]. Based on anatomy and neural tuning, the frog TS exhibits similar evidence for convergence, with extensive complex sensitivity, including stimulus combination sensitivity, integrated frequency channels, and temporal tuning [18, 46–52]. With regard to FM stimuli, however, there are relatively few tests of single neuron sensitivity in the frog TS compared to those in mammals [40]. Ponnath et al. [50] found evidence for frequency specific adaptation in phasic TS units: compared to the phasic response to tones, FM stimulation elicited a more tonic response due to the change in frequency in the FM stimuli. In experiments using more functionally relevant sounds, TS neurons in *Eleutherodactylus coqui* exhibit different excitatory responses for upward and downward FM sweeps [53]. Neither of those datasets, however, investigated the extent to which individual cells code the specific FM parameters required to elicit behavior. In contrast to single cell recordings, multiple studies have taken an indirect approach to evaluate TS coding of species-specific FM parameters in anatomical nuclei. For example, in response to several minutes of acoustic stimulation, immediate early gene (IEG, *egr-1* mRNA; also called *ZENK*, *zif*268, *NGFI*-A, and *krox*-24) induction shows that cells in all TS subdivisions in túngara frogs exhibit increased response to stimuli containing the FM whine [54]. These IEG responses are indicative of neuronal excitability [55] and could predict action potential responses in individual TS neurons increase with stimulation by the parameters in the whine’s FM [30]. By using many of the same stimuli generating the model by Wilczynski et al. [30], we tested whether excitation in isolated TS units is indeed tuned to whine stimulation and if that tuning is based on the same FM transition critical to behavior. Additionally, we use signal detection theory to assess whether action potential responses to the FM transition is sufficient to account for behavioral discrimination of whine and noise, the phonotactic test that determined the information-bearing parameters in the FM whine [30].

## Materials and methods

### Animals

Túngara Frogs, *Physalaemus pustulosus* (N = 31; yielding 110 analyzed cells; females yielded 79% of cells) were colony reared (University of Texas-Austin) from founders collected in Gamboa, Panama.

### Ethics statement

All animal care and experimental procedures were performed in accordance with and approved by the institutional animal care and use committee (IACUC) at Louisiana State University Health Sciences Center (IACUC #3542) and the University of Texas-Austin (AUP-2015-00051).

### Preparation and recording

The protocol for extracellular recordings of isolated TS units in awake túngara frogs is similar to that used previously [49, 50]. Under general (SQ injection MS-222; 0.16 mg/g) and topical anesthesia (dibucaine cream; 0.9%), the midbrain was exposed by resecting a piece of skull dorsal to the optic tectum. After 24 h of recovery from anesthesia, immobilized frogs (i.m. succinylcholine chloride; 22 μg/g body weight; [56]) were mounted dorsal side up on an air table in a foam (Tecnifoam 4 in.; NRC 1.21 at 500 Hz) lined Faraday cage and kept moist to maintain cutaneous respiration. All recordings were carried out at 19.5–22.5°C. Extracellular electrophysiological activity was recorded using thin-walled glass micropipettes filled with 4 M NaCl (3–10 MΩ). After amplification (GRASS P511 with high impedance head stage), neural responses were digitized (100 μs sampling period) using a TDT AD3 and System II array processor with custom written software. TS auditory units were isolated (peak action potential voltage was >20 dB above the noise at recording start) using a series of search stimuli covering the frequency range of auditory sensitivity and call spectra (~0.1–5 kHz) [57]. These stimuli included a 60-ms Gaussian noise, 30-ms tones, and a 20-ms band limited noise centered at 2 kHz. Call stimuli were not used in the search to avoid any potential of adaption to test stimuli. The recording site was recovered using the stereotactic position of the electrode, which was calibrated by current injection lesion and nissl stain [49, 50]. Electrodes were inserted in the central and medial portions of the tectum with recording sites ranging across the dorsal-ventral depths of the midbrain, with most recordings in the principle nucleus of the TS.

### Acoustic stimulus production

Acoustic stimuli were generated and amplified using a TDT II DA3 16 bit D-A converter (40 μs sample period) and a Harmon/Kardon integrated amplifier, respectively. Stimulus amplitude was controlled with TDT PA2 programmable attenuators. Acoustic stimuli were presented from a single Fostek #FE127 broadband speaker positioned at 0° normal to the front of the frog (30 cm distance). The speaker was calibrated at the position of the recording site, directly between and dorsal to the two tympana using a Bruel and Kjaer B&K 2608 measuring amplifier with a B&K model 4133 1/2-in. microphone and a B&K 4220 pistonphone calibrator. All frequency components of the chamber’s ambient noise were ≤ 21.5 dB SPL (peak ambient noise range 120–230 Hz).

### Frequency tuning and relative sensitivity to call parameter stimuli

Isolated cells were used in one or both of two experiments. Experiment 1 determined single unit frequency tuning from 0.2 and 5.1 kHz (50 or 100 Hz steps) using 200 ms tone pulses (1 ms cosine ramps) and an adaptive procedure (≥3/5 down, ≤2/5 up; ±3 dB resolution). Subsequently, the flanks of the tuning curves were modeled using a least-squares fit of a rounded exponential filter function. Functions with significantly fitting (P<0.05) enabled calculation of the equivalent rectangular bandwidth (ERB) of the tuning curve filters [15, 50, 58–60]. With respect to descriptive measures of frequency tuning, two sample t-tests were used to compare the difference in the means of ERBs and the best frequency thresholds for the low and high frequency (AP and BP) channels.

**Fig 1:**
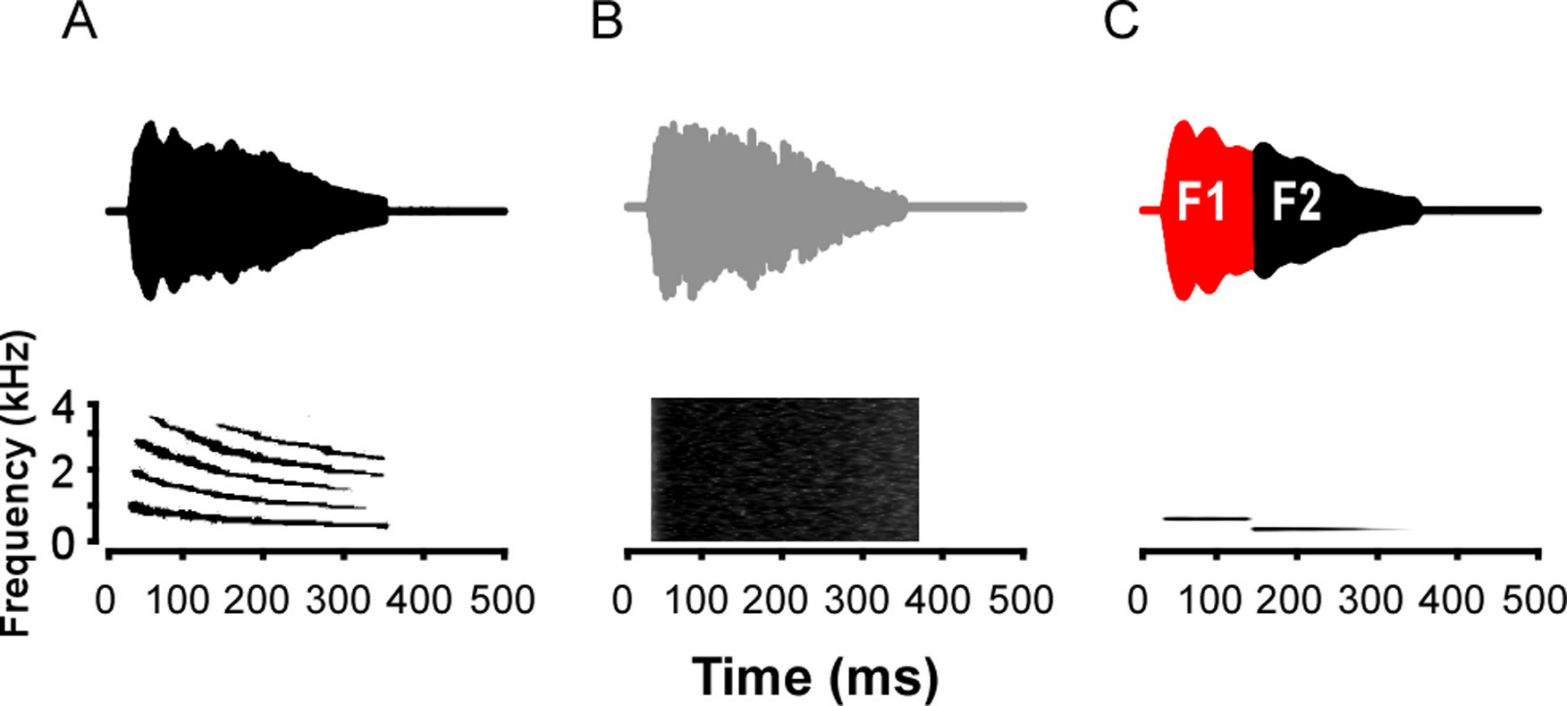
Examples of stimuli. Upper and lower rows are time waveforms and spectrograms, respectively, for three of the stimuli used to test auditory sensitivity in isolated units of the mid-brain. (A) whine; (B) broadband noise with whine envelope; (C) two tone FM step (800 to 500 Hz) with whine envelope.

Experiment 2 was modeled after the one used to assess which acoustic parameters of the whine elicit phonotaxis [30]. The experiment only included whine sensitive units: after recording isolation using the search stimuli, several natural whine stimuli (see below) were presented. If action potentials were elicited, then the battery of test stimuli ensued. The envelope and duration (325 ms) of all test stimuli were those of the natural túngara whine call (or time-reversed; see below), which was calculated (Hilbert transform; [59]) and smoothed using a Savitzky-Golay FIR smoothing filter (span 81, degree 5) in MATLAB. The following are the 14 test stimuli (examples in Fig 1). The natural whine: recorded from one of 50 calling males in Gamboa, Panama, it is the call closest to the population mean of 15 acoustic variables [61]. This whine has been used previously to elicit robust female phonotaxis [20, 22, 62, 63]. Reverse whine: the time-reversed natural whine. Noise: broadband noise with the whine envelope. Reverse noise: broadband noise with time-reversed whine envelope. Single tones at frequencies that are components of the dominant frequencies of the FM whine: 430, 500, and 800 Hz. Sequential frequency transitions: consist of two sequential tones with an instantaneous frequency step at a sinusoidal zero crossing, which prevented spectral splatter. The transition (step) times match the relative times used in behavioral tests [30]. The time is noted relative to stimulus onset: 900 to 430 Hz (162 ms), 430 to 900 Hz (162 ms), 800 to 500 Hz (109 ms), 500 to 800 Hz (109 ms), and 700 to 550 Hz (109 ms). Because the natural call used here was slightly longer than the synthetic one used by Wilczynski et al. [30], these transition times are slightly delayed (~10 ms) from theirs. Simultaneous tones: concurrent presentation without FM step of 900+430 Hz or 800+500 Hz. Stimuli were presented in pseudorandom order, with whine and reverse whine always tested in the first two blocks before moving to the other 12 stimuli. This ensured that responses to these first stimuli were recorded, as they form the basis of the analysis of sensitivity both to the FM transition parameters and the effects of envelope shape. All stimuli were presented in 20 repetition blocks with 2 s stimulus period. As part of signal detection theory analysis (see below), we assessed how responses correlated to particular ongoing FM stimulus components. Thus, stimulus spectrograms were calculated using short-time Fourier transforms (MATLAB). The dominant frequency in the natural whine was determined from the power spectral density matrix of the spectrogram as the frequency component with the highest amplitude.

After isolating a unit, the order of the two experiments (tuning curves using tones; series of complex FM stimuli) was pseudo-randomized. Depending on the ability to hold the isolated recording, some cells were tested in both experiments and others in only one. Typical of midbrain recordings, success rate (number of isolated units) varied between subjects, with some frogs yielding more units than others. Similar to work using *in vivo* recordings in frog TS, we have pooled units across subjects to understand the variance in processing capabilities at this auditory nucleus [49, 50, 64–67].

### Analysis of responses to varied call parameters

Responses to stimuli with varied call parameters were quantified as the mean number of spikes per stimulus for 20 repetitions. The capture buffer duration was 1000 ms. Stimulus driven responses within cells were corrected for spontaneous activity by subtracting the cell’s mean spontaneous rate (mean of 20 repetitions of silence [49]). Note that this correction was minimal because spontaneous activity was low, as only two cells showed >2 spontaneous spikes per ‘silent’ sweep (they were 2.2 and 4.7 spikes/silent stimulus presentation).

For each cell, responses to the different stimuli represent repeated measures. Additionally, the design is unbalanced, as the number of stimuli per cell differed because not all cells completed recordings for all test stimuli. Thus, a linear mixed effect model is fitted to compare whether excitation differed between the battery of test stimuli. The model has a random intercept to account for the variation across cells. With covariates, sex, stimulus ID, and their interaction, we chose compound symmetry (CS) covariance structure due to the facts that convergence criteria were not met with unstructured covariance, and that it is not reasonable to assume a covariance structure other than CS because there is not a natural order in stimuli. Furthermore, with AR(1) covariance structure (autoregressive), the Akaike Information Criterion and Bayesian Information Criterion are about the same as those using CS structure. The variance of the random intercept is significantly different from zero (p = 0.0008), and the covariance in CS structure is significantly different from zero (p < 0.0001). With this model, we concluded that both sex (p = 0.8962) and the sex*stimulus ID interaction (p value = 0.5655) have negligible effect and were removed from the model. The responses of cells from males and females are thus pooled to compare stimulus responses.

To compare the mean responses to the whine and those to the other test stimuli, we obtained Huber robust estimates of standard errors, and then used Dunnett-Hsu method to adjust p values for multiple comparison correction with whine as the control. The whine is used as the standard in all but one comparison because the whine is already known to contain the information-bearing element (in the form of FM) necessary and sufficient to elicit phonotaxis [30]. The comparison between noise and reverse noise was carried out separately by using a contrast test. All the computation was carried out in SAS 9.4 (Cary, North Carolina, USA), employing the MIXED procedure.

Note that the use of 20 repetitions for each stimulus creates a potential effect of adaptation. Thus, repetition number was analyzed by a nonparametric Friedman test and associated rank sum with correction for multiple comparisons. The test evaluated if the repetition number (1 to 20) was associated with the amount of excitation.

## Results

**Fig 2:**
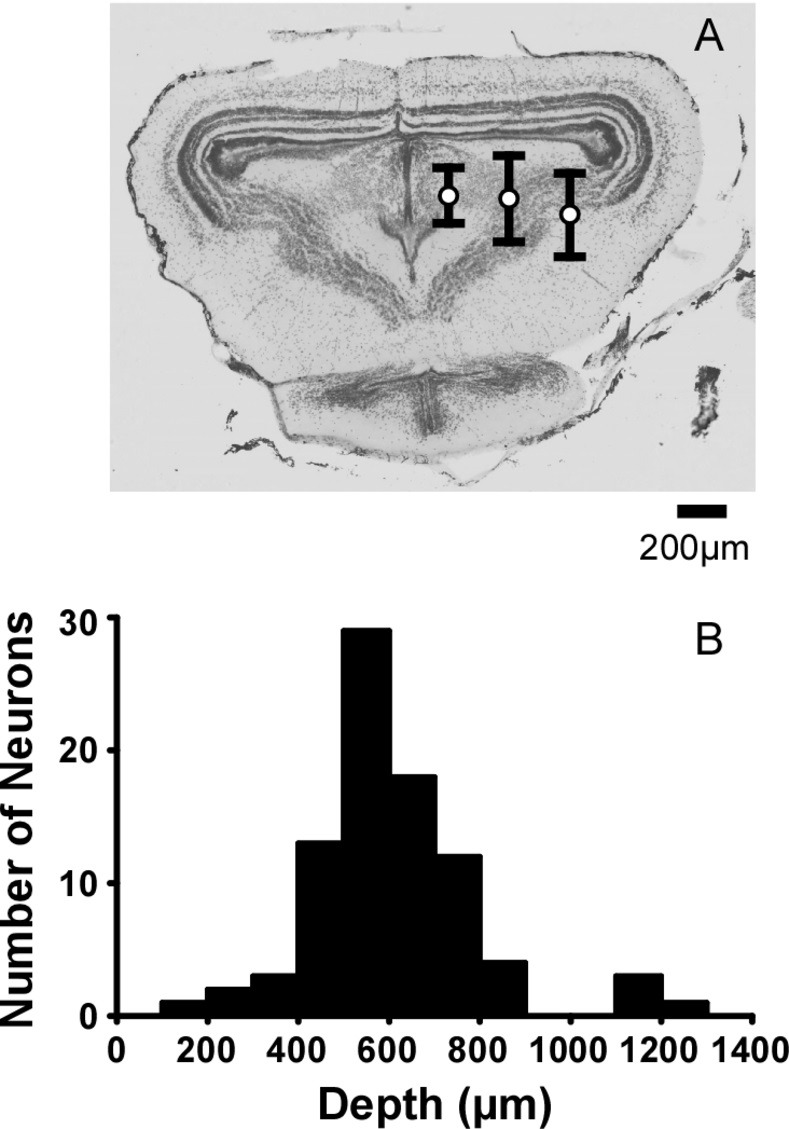
Distribution of recording positions. (A) Mean (±s.d.) depth for recorded cells. The electrode positions are categorized into three penetration columns: the medial, central, and lateral one thirds of the midbrain. The positions are plotted on a transverse section of the midbrain (upper schematic) stained with cresyl violet. (B) Histogram of depth for all whine sensitive cells, showing most recordings 500–700 μm deep, bin width 100 μm.

The search stimuli enabled isolation of 110 units. The sample sizes for tests of frequency tuning to tones and call parameter stimuli were 65 and 87 cells, respectively. Whereas 42 cells contributed to both experiments, 23 contributed only to frequency tuning to tones and 45 cells contributed only to call parameter testing (yielding 110 total cells). The distribution of recording positions primarily covered areas known to contain auditory cells [54, 68]; most were located in nuclei of the torus, but also included some positions in the optic tectum and tegmentum (Fig 2).

### Frequency tuning characteristics

**Fig 3:**
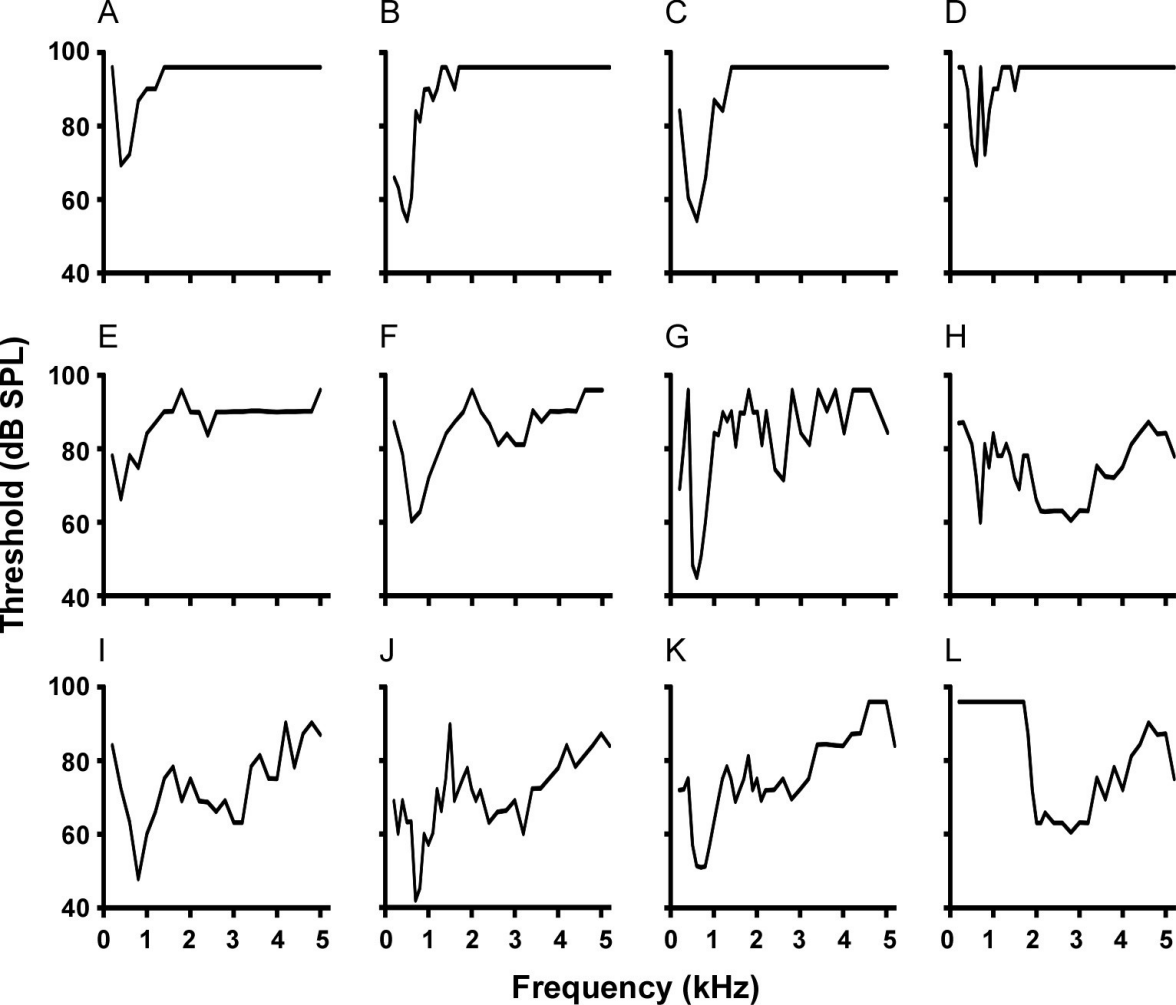
Example tuning curves showing cells with best frequencies ranging from low to high frequencies in panels A-L, respectively. (A-D) Cells exhibiting low frequency tuning only (<1.2 kHz), potentially receiving input from the amphibian papilla. (E-K) Cells exhibiting ‘W’ shaped tuning, potentially corresponding to input from both the amphibian and basilar papillae. (L) Example of high frequency (>1.2 kHz), potentially receiving input from the basilar papilla, only. This cell (L) contributed to the analysis of frequency tuning, but did not respond to the whine.

**Fig 4:**
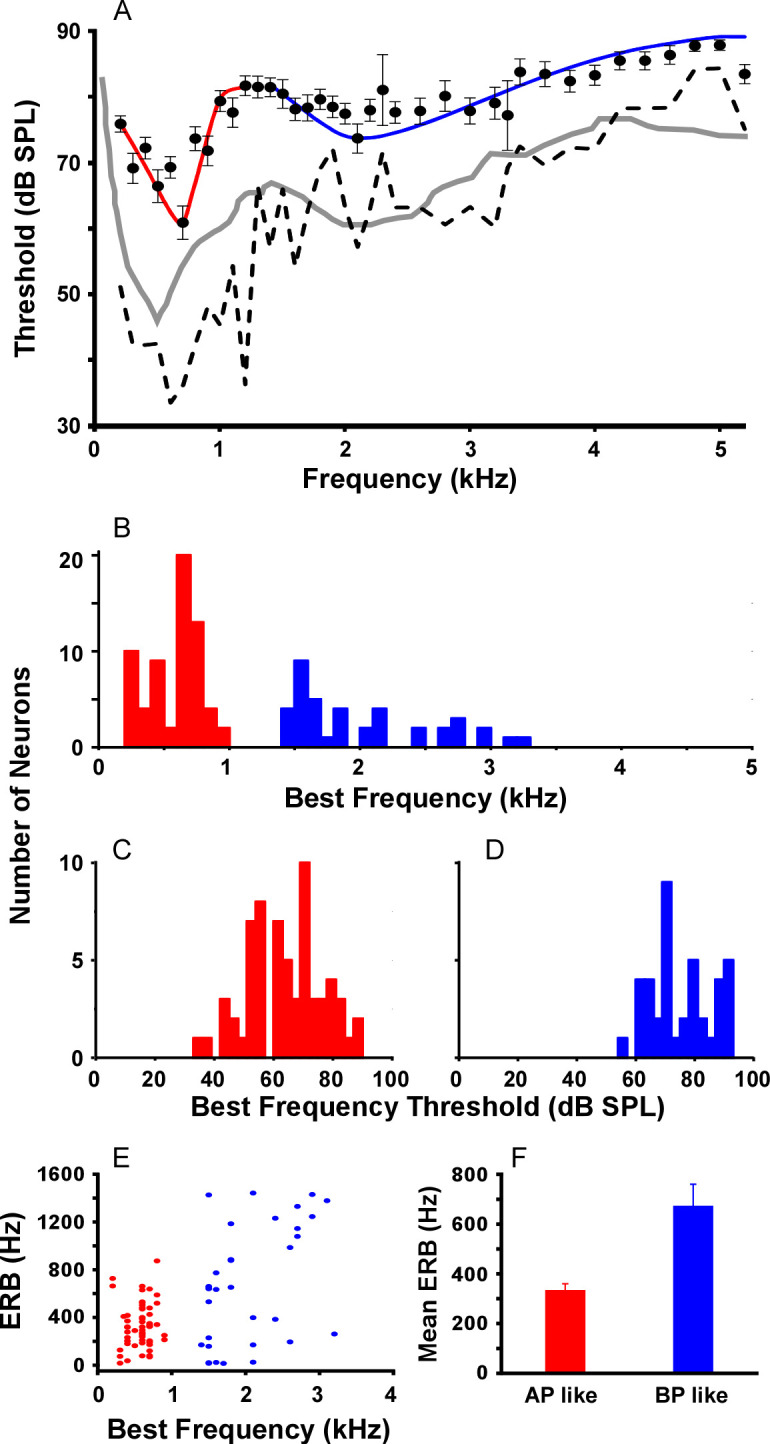
Summary of frequency sensitivity characteristics of cells. (A) Overall tuning from all recordings. Filled circles are the mean (±s.e.) threshold for each frequency. Red and blue curves are the least squares fits of the rounded exponential function to the mean thresholds for the low and high frequency channels (1.2 kHz boundary), respectively [69]. Dotted line shows the lowest threshold at each frequency across all cells. Grey curves are previous threshold data measured using multiunit recordings from large areas of the TS from Ryan et al. [69]. (B) Histogram showing the distribution best frequencies in the low and high frequency channels, revealing two distributions with no best frequencies from 1–1.4 kHz (histogram bin width = 100 Hz). (C-D) Histograms showing the distribution of best thresholds at frequencies below and above 1.2 kHz (blue and red, respectively). (E) Relationship between best frequency and equivalent rectangular bandwidth (ERB) size. Red and blue are ERBs centered at best frequencies below and above 1.2 kHz, respectively. (F) Mean (±s.e.) ERB size in the low and high frequency bands (amphibian papilla, AP and basilar papilla, BP).

Data here are the first to show single unit recordings in the túngara frog midbrain, as previous work used gross multiunit recordings [69]. Frequency tuning curves were measured using tones in 65 cells, with best sensitivity represented by three general shapes: low frequency only (<1.2 kHz; 25 cells)(Fig 3A–3D), high frequency only (>1.2 kHz; 1 cell)(Fig 3L), and low-high frequency or a combination of the two bands, creating ‘W’ shaped tuning curves (39 cells)(Fig 3E–3K). The latter shape means cells can have two sensitivity peaks (i.e., two ‘best’ frequencies), allowing for analysis of their contribution to low and high frequency band sensitivity. Averaging all tuning curves reveals the contribution of these two sensitivity bands to overall tuning (Fig 4A), likely reflecting integration of peripheral input from the amphibian (AP) and basilar papillae (BP) (low and high frequency channels, respectively; [69]). The boundary of the two bands, measured as the highest midpoint threshold between the two lowest thresholds, is 1.2 kHz. The low frequency band (best frequency at 700 Hz) was more sensitive than the high frequency band (best frequency at 2.1 kHz) with mean (±s.e.) best frequency threshold of 60.8 (±2.55) and 73.6 (±2.21) dB SPL, respectively (Fig 4A). There was extensive overlap of tuning in the low frequency band, with most cells exhibiting best frequencies near 700 Hz. In contrast, best frequencies across cells in the high frequency band were more broadly distributed (Fig 4B). Both bands exhibited a wide range of thresholds at the best frequencies, ranging over 30–40 dB (Fig 4C and 4D). Fitting the low frequency (AP) and high frequency (BP) components of each cell’s tuning curves with a rounded exponential filter function enabled calculation of equivalent rectangular bandwidth (ERB). ERB of tuning was narrower in the low frequency than the high frequency channel (Fig 4E–4F; ERB Low = 334.7 ±25.4 Hz, N = 55; High = 674.3 ±86.0 Hz, N = 33, t-test; p<0.00053), raising the possibility of a positive correlation between BF and ERB [70]. When including low and high frequency BFs, linear regression analysis revealed such a relationship (N = 88 ERBs, Coef. = 0.257, intercept = 172.8 Hz, R^2^ = 0.303; p<0.0000001). However, the relationship was driven by the wide bandwidth data from the BP-like band. If those best frequencies and ERBs are removed so that regression only includes ERBs with BFs <1.2 kHz, then there is no significant BF vs. ERB correlation in the AP-like channel alone (N = 55, Coef. = 0.104, intercept = 274.7 Hz, R^2^ = 0.008, p = 0.507). Note, as described above, the single cell exhibiting only high frequency sensitivity (Fig 3L) was unusual in our sample. It was not sensitive to the whine and thus not included in the analysis of FM sensitivity. Finally, with respect to the relationship between frequency sensitivity and anatomy, there was no correlation (linear regression) between recording depth and the single best frequency for each cell’s tuning curve (R^2^ = 0.00109; p = 0.846).

### Response to varied call parameters

**Fig 5:**
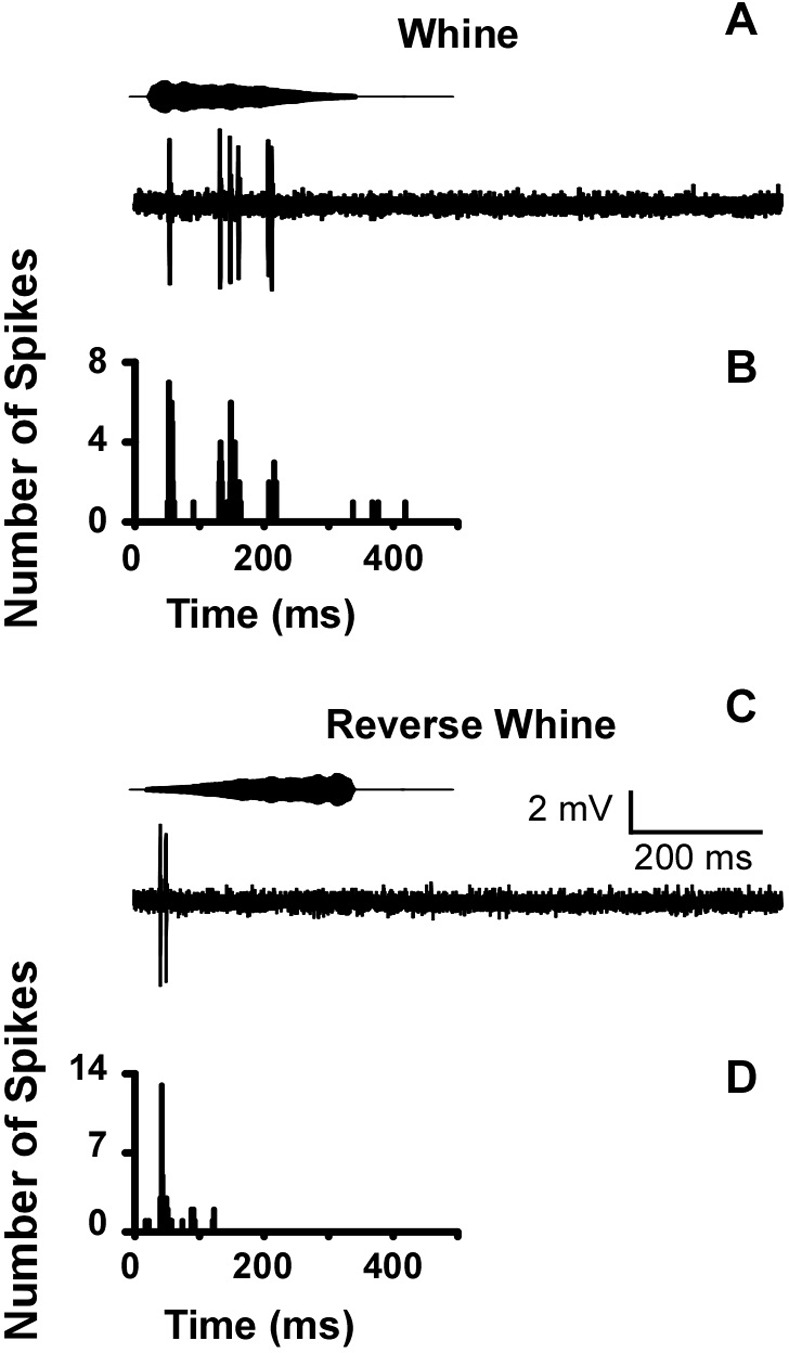
Example of a whine sensitive cell with reduced sensitivity to reversed whine. Panels are spike trains in responses to single presentations and peristimulus time histograms for 20 repetitions of the (A, B) natural whine and (C, D) reversed whine (time-waveforms of stimuli above voltage traces).

**Fig 6:**
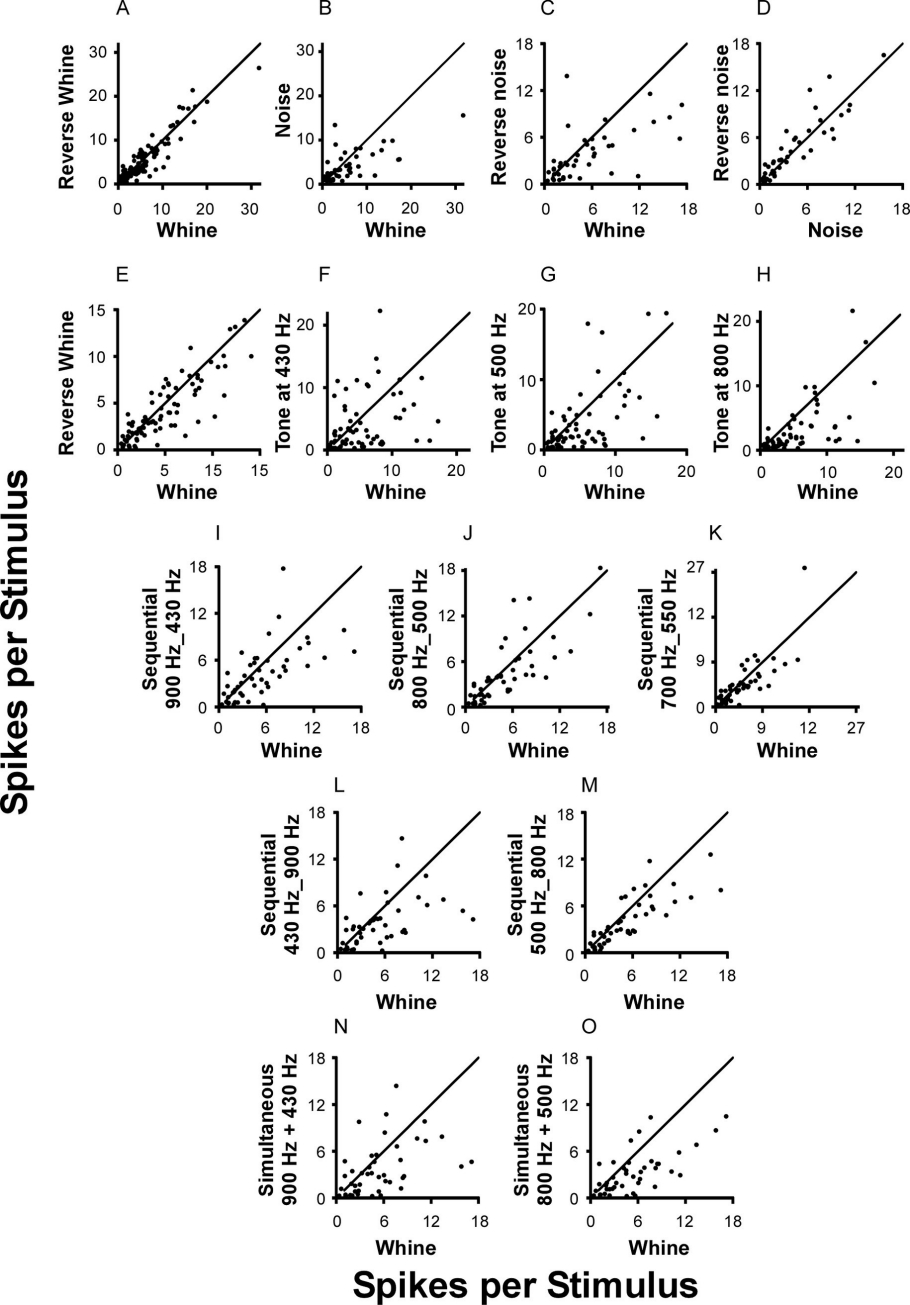
Graphic comparison of each cell’s response to call stimuli with varied parameters. Each point is the mean number of spikes per stimulus for the two stimuli shown on the X and Y axes. In all but panel D, the Whine is the reference stimulus. Lines are that predicted if the responses are equal. Note that panel E is an expansion of the axes in panel A so that data close to the origin can be viewed.

Using tones to measure tuning in the previous dataset does not allow for assessment of whether modulation in stimulus spectrum alters excitability. Due to its effectiveness in eliciting phonotaxis, the natural whine, an FM signal, was used as a standard for comparisons of the effect of frequency modulation on spike count. In general, stimuli with descending FM, especially the natural whine, but including downward tone steps, were most excitatory (Table 1). The whine elicits significantly greater responses than nearly all stimuli even when the spectral content was the same. For example, time-reversing the whine, which changes the temporal direction of the FM and envelope, significantly reduces the spike response (e.g., Fig 5). The whine is also more excitatory than tones and ascending FM steps. But, interestingly, it was not as strongly different from descending FM step stimuli (Table 1). In particular, response to the 700 Hz to 550 Hz step is not significantly different. Finally, spike number is not affected by reversing the envelope shape, which was isolated by comparing responses to noise with the natural whine envelope to responses to noise with the envelope in reverse. Different from reversing the temporal sequence of FM, as in the reverse whine, the use of noise removes frequency modulation while keeping the broad spectrum (Table 1). Graphic representation of within-cell comparisons of spike count are presented in Fig 6.

**Table 1:** Linear mixed model analysis of spike response to whine or noise versus experimental stimuli. Columns are: the standard stimulus (basis for comparisons) and the mean spike number (SEM); the alternative stimulus and mean spike number (±s.e.); number of cells in the dataset recorded for both stimuli; difference in spike number, the adjusted p value of the comparison, and the discrimination index (d_a_) for the entire stimulus response. Graphic representation of the within-cell comparisons are in Fig 6.

Standard Stimulus	Estimate Mean Spikes per stimulus	Alternative Stimulus	Estimate Mean Spikes per stimulus	N Cells in comparison	Estimate of difference	Adjusted p value	Overall Discrimination index (d_a_)
Noise	3.95 (0.47)	Time reverse noise	4.15 (0.52)	42	-0.20 (0.25)	0.4377	0.030
Whine	6.24 (0.58)	Reverse whine	5.44 (0.57)	87	-0.80 (0.22)	0.0031	0.150
		Noise	3.95 (0.47)	45	-2.29 (0.57)	0.0012	0.470
		Time reverse noise	4.15 (0.52)	42	-2.09 (053)	0.0011	0.490
		**Single Tone**					
		430 Hz	4.41 (0.62)	63	-1.83 (0.64)	0.0407	0.375
		500 Hz	4.19 (0.60)	63	-2.05 (0.51)	0.0007	0.419
		800 Hz	3.44 (0.54)	59	-2.80 (0.46)	<0.0001	0.631
		**Descending Sequential Tones**					
		900 Hz_430 Hz	4.67 (0.50)	49	-1.57 (0.43)	0.0034	0.367
		800 Hz _500 Hz	5.00 (0.55)	47	-1.24 (0.40)	0.0189	0.234
		700 Hz _550 Hz	5.04 (0.62)	45	-1.20 (0.44)	0.0625	0.228
		**Ascending Sequential Tones**					
		430 Hz _900 Hz	4.13 (0.47)	47	-2.11 (0.47)	<0.0001	0.514
		500 Hz _800 Hz	4.39 (0.44)	47	-1.85 (0.32)	<0.0001	0.439
		**Simultaneous Tones**					
		900 Hz + 430 Hz	4.06 (0.53)	44	-2.18 (0.52)	0.0004	0.528
		800 Hz + 500 Hz	3.56 (0.43)	44	-2.69 (0.39)	<0.0001	0.720

**Fig 7:**
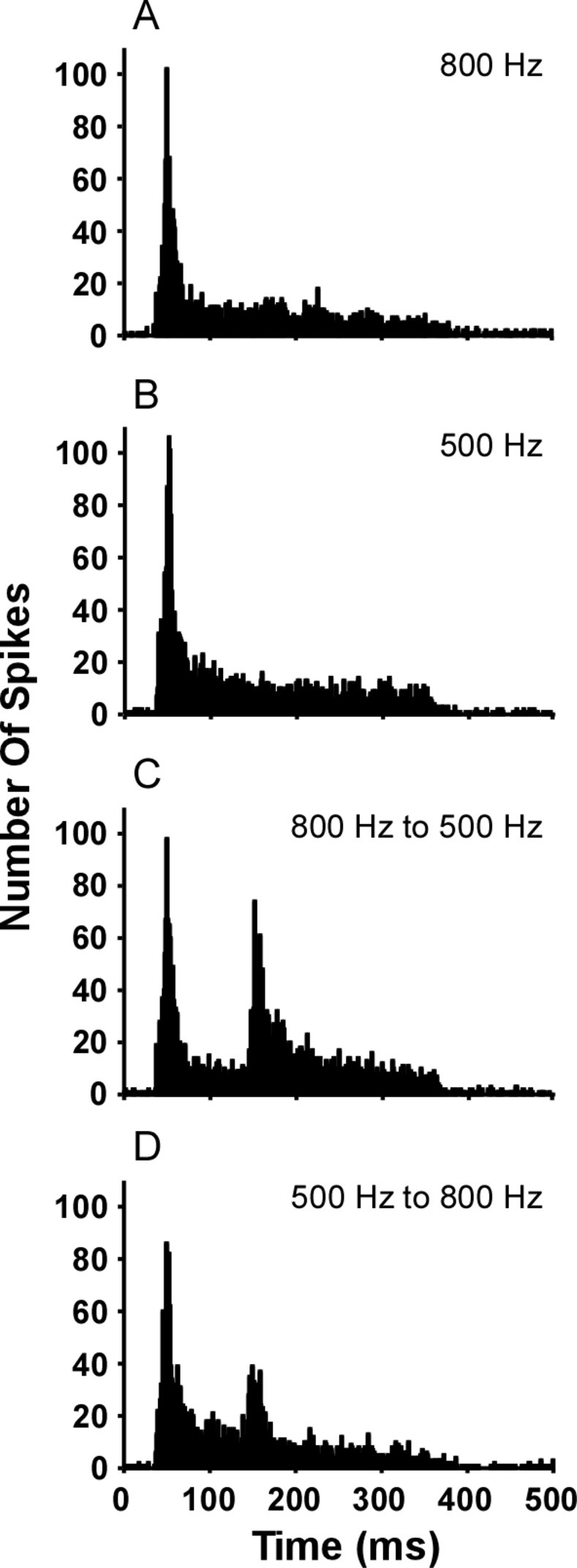
Peristimulus time histograms for responses of cells to tones and FM steps. Each cell completed 20 repetitions of the four stimuli, presented in pseudorandom order. Stimuli were: (A) 800 Hz tone; (B) 500 Hz tone; (C) 800 Hz to 500 Hz step FM; D) 500 Hz to 800 Hz step FM. Downward FM elicits a large mid-stimulus response, less so for upward FM, and not evident for tones. All stimuli had 325 ms duration and the natural whine envelope (Fig 1). The FM step transition is 109 ms after stimulus onset (at 25 ms). Histogram bin width is 1 ms. N = 52 cells.

How is the greater excitation to the whine (and potentially whine-like FM) manifested in the spike train? Fig 7 shows evidence for increased excitation associated with an FM transition (appearing as a histogram ‘bump’). This increased excitation or ‘bump’ could be functionally relevant: in behavioral choices against noise, whereas the 800 Hz to 500 Hz step elicits whine-like preferences, 800 Hz alone, 500 Hz alone, and the 500 Hz to 800 Hz stimuli do not [30]. Thus, subsequent analysis determined: whether such a ‘bump’ occurs in response to the whine (rather than an FM step stimulus), what frequency transition in the whine creates the largest histogram ‘bump’, and if that FM transition matches that critical to behavior [30]. Finally, signal detection theory was used to compare histograms to determine if the discrimination index value of the ‘bump’ is sufficient to explain discrimination of whine (signal) vs. noise.

### Signal detection theory analysis of responses to whine vs. noise

**Fig 8:**
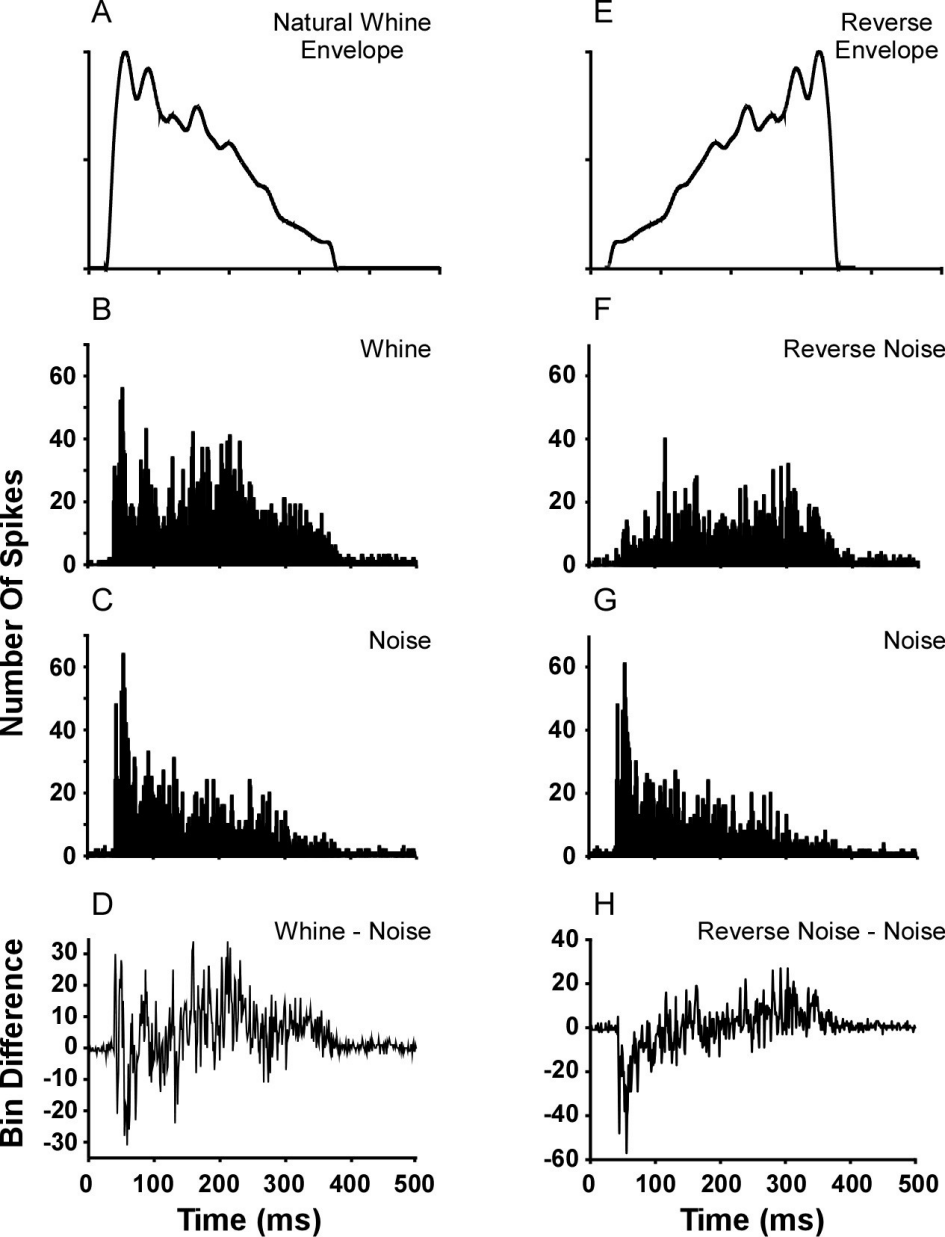
Comparison of peristimulus time histograms for whine versus noise and reverse noise versus noise. Stimuli in left column have the natural whine envelope, shown rectified in (A). (B-C) Histograms of spike number for whine and noise stimuli, respectively (N = 45 cells). (D) Per bin difference between whine and noise histograms, revealing whine greater than noise response between ~150–250 ms. Right column: test of whether increased histogram response (~150–250 ms) can be generated by amplitude alone. (F-G) Histograms of mean number of spikes per bin for reversed noise and noise, respectively (N = 45 cells). Whereas the noise has the natural whine envelope (A), the envelope is reversed in reversed noise (E). (H) Per bin difference between reversed noise and noise histograms fails to generate an increased difference in spike number even with increasing envelope.

**Fig 9:**
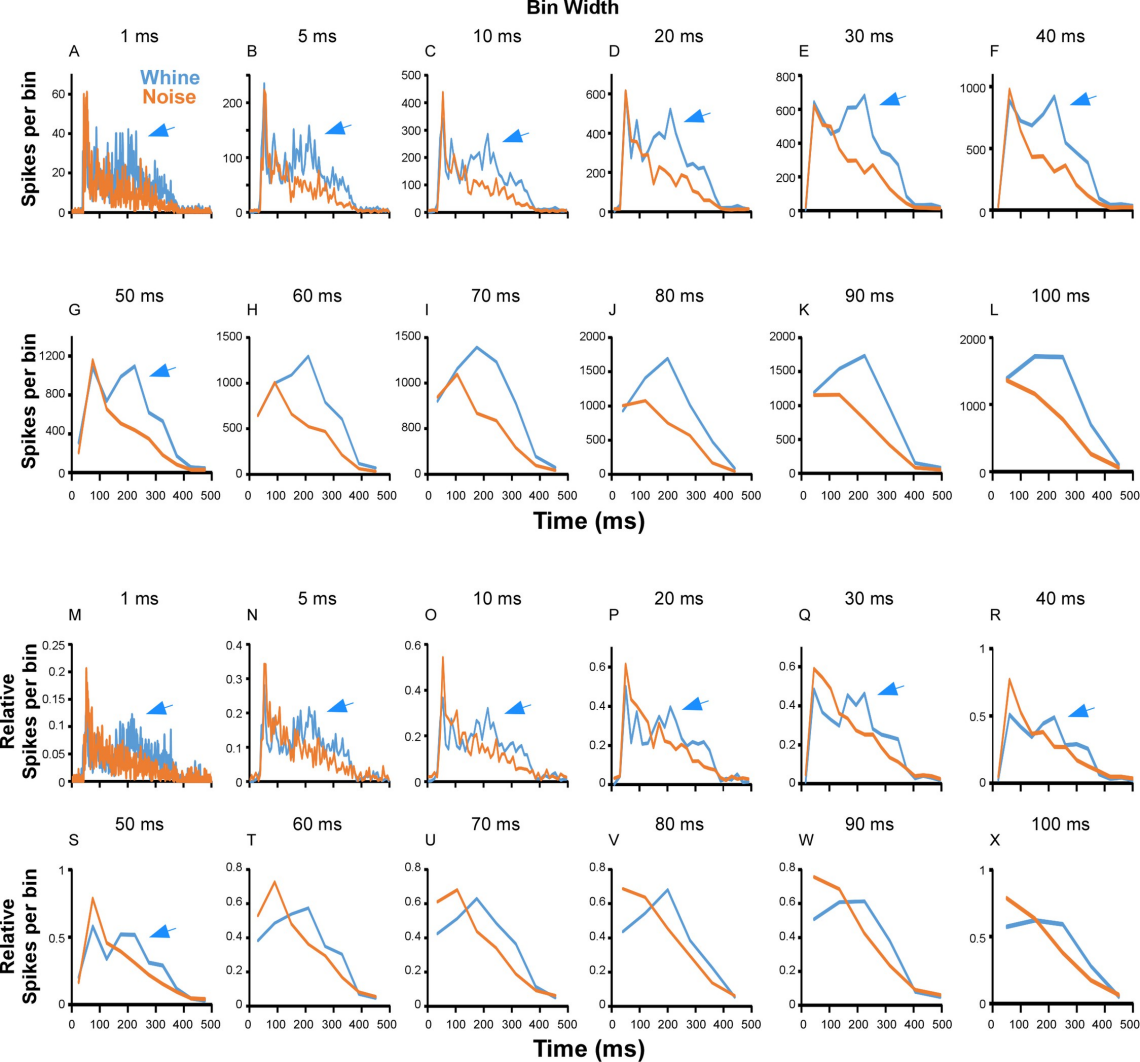
Effect of bin width on histogram shape. Each panel shows the peristimulus time histogram responses to the whine (blue) and noise (red) summed across cells (N = 45 cells). Bin width is noted above each panel. (A-L) Y-axis shows the number of spikes per bin. (M-X) Y-axis shows the mean relative number of spikes per bin. The mean is calculated after each cell’s histogram is normalized to its maximum bin count. After the initial burst, the increased response to the whine between ~150–250 ms (blue arrows) is evident for bin widths up to 50 ms and not an artifact of bin width.

The mixed model analysis assesses the overall response (i.e., number of spikes in the recording buffer) to stimuli and does not address whether a particular part of the histogram, and thus part of the stimulus, is responsible for the whine’s dominance in Table 1. However, combining the responses of all cells to create an experimental multi-unit response (with mean and variance) in a single spike histogram enables calculation of an ongoing discrimination index for two stimuli (e.g., d’). Spike times for all cells (N = 45) that completed stimulation by the natural whine and noise were binned in peristimulus time histograms (i.e., one histogram each for whine and noise; Fig 8B and 8C). Each bin represents the mean number of spikes to the whine or noise. Taking the difference in the histograms (Fig 8D) reveals an increase in spikes during the 150–250 ms time frame of the whine, like the phenomenon found for FM steps (Fig 7). The whine elicits an initial burst of spikes (first ~40 ms of stimulus), followed by a slight decrease. Subsequently, a large ‘bump’ or increase occurs (at 150–250 ms), followed by a decay. In contrast, the noise stimulus elicits an initial burst followed by a decay. The call envelope (Fig 8A) appears to explain the initial burst, as reversed noise, which lacks the high amplitude beginning, does not elicit the same initial response (Fig 8E–8G). Furthermore, response to the reversed noise also shows that the ‘bump’ in the whine histogram cannot be produced by a rising envelope, as the reversed noise’s increasing amplitude is unable to elicit a large secondary ‘bump’. It is also important to note that the ‘bump’ or spike increase for the 150–250 ms time frame of the whine is not an artifact of a particular histogram bin width. Whether the histogram is plotted as absolute or relative (to each cell’s max bin count) mean number of spikes/bin, the ‘bump’ remains for bin widths from 1 ms to ~50 ms (Fig 9). No such ‘bump’ occurs for noise, as those histograms follow the stimulus envelope, regardless of bin width (Fig 9).

Although the absolute difference between the values in each bin (mean spike number) in the whine and noise histograms shows several large differences (especially in the 150–250 ms range; Fig 8D), a relative difference is required to determine the extent to which the ‘bump’ is sufficient to discriminate whine (signal) from noise responses. Thus, using the relative bin counts (Fig 9) we calculated an ongoing (for each bin) discrimination index that accounts for potential unequal variances in each bin’s spike response: the difference in mean response per bin (whine bin–noise bin) divided by the average variance of the two bins [71–73]. This index, referred to as d_a_ (rather than d’), enables the calculation of the per bin discrimination index for the two stimuli.


$$Per\;bin\;d_{a}=\frac{\mu_{whine}-\mu_{noise}}{\sqrt{\frac{\sigma_{whine}^{2}+\sigma_{noise}^{2}}{2}}}$$

**Fig 10:**
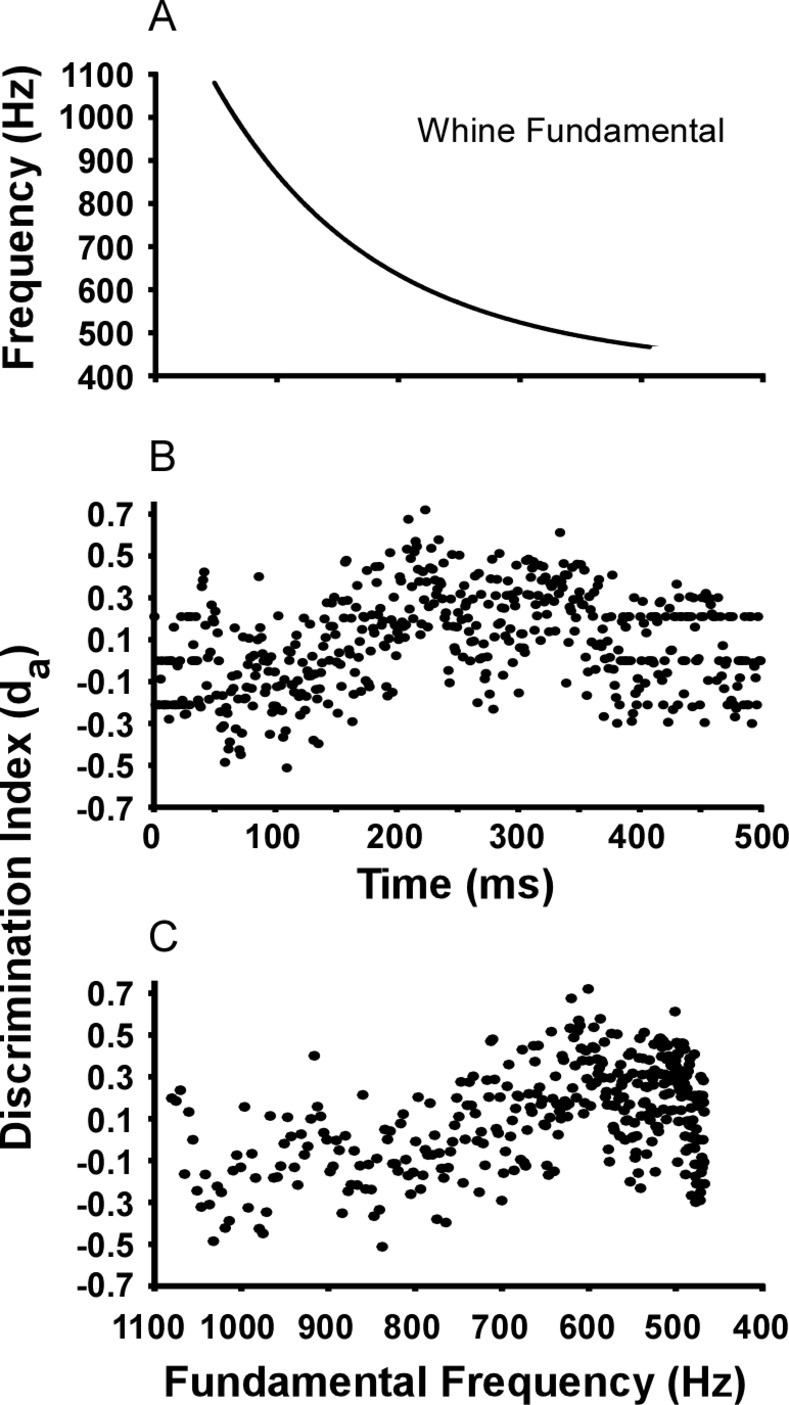
Whine versus noise ongoing discrimination index (d_a_) as a function of time and whine fundamental frequency. (A) Fundamental frequency of the natural whine stimulus used in these experiments. (B) Ongoing discrimination index as a function of buffer time. (C) Same discrimination index after stimulus time was converted to whine fundamental frequency. Peak d_a_ occurs as the fundamental passes through 601 Hz (calculated using 1 ms bin width).

**Fig 11:**
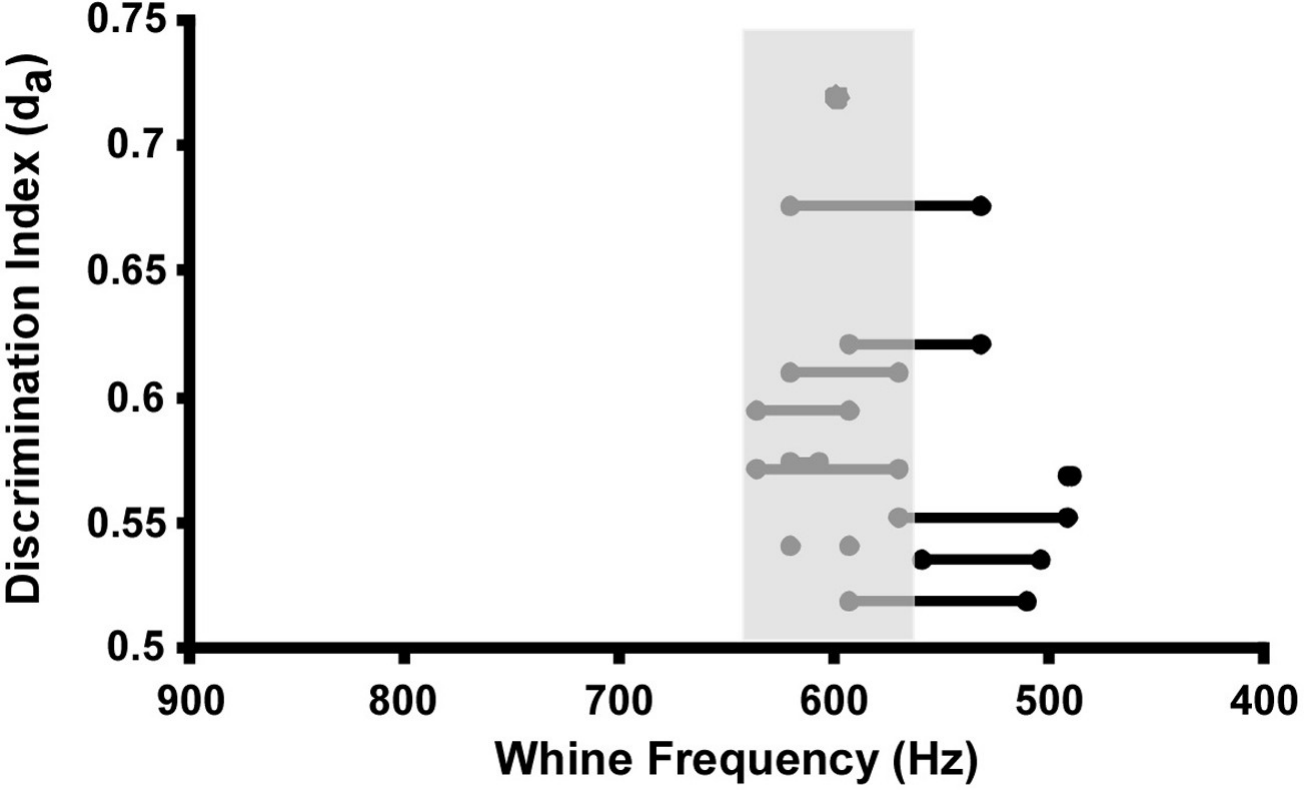
Whine fundamental frequencies eliciting highest discrimination of whine versus noise when calculated using different bin widths. Symbols (also connected by lines) show the d_a_ for bins with maximum sensitivity when plotted on the x-axis not as bin width (time), but as whine frequency. The 1 ms bin width yielded the highest d_a_ at 601 Hz. Grey area represents the transition frequencies important to whine recognition, which were predicted by Wilczynski et el. [30] from behavioral data.

Using a 1 ms bin width, the large ‘bump’ in the whine response histogram generates a peak in discrimination when compared to the noise response. Using the mean first spike latency (23.5 ms) to sync bin responses to the whine, the maximum d_a_ (0.721) occurs at the time when the whine fundamental frequency is 601 Hz (Fig 10). Thus, FM transition through this frequency elicits the largest discrimination against noise, predicting 69–70% behavioral choice of whine over noise. Note that the increased number of action potentials near the 600 Hz portion in the whine’s FM transition cannot be explained by the spectral tuning of the cells. Many cells are indeed tuned to frequencies near 600 Hz, potentially increasing responses as the modulated fundamental ‘passed through’ this low threshold area of tuning curves. However, such an effect would be equally likely for the reverse whine and reversed step FM stimuli. This increase in spike number was not found, and thus more consistent with downward FM sensitivity. It is important to point out that there is no biological justification for using 1 ms bins in this analysis. This was chosen to provide the sharpest resolution of stimulus frequency at the highest d_a_. Fig 9 suggests that other bin widths may yield similar stimulus times of high discrimination. When d_a_ is calculated at other bin widths, although reduced in value, the times of highest discrimination largely overlap the behaviorally relevant FM transition around 600 Hz (Fig 11).

**Fig 12:**
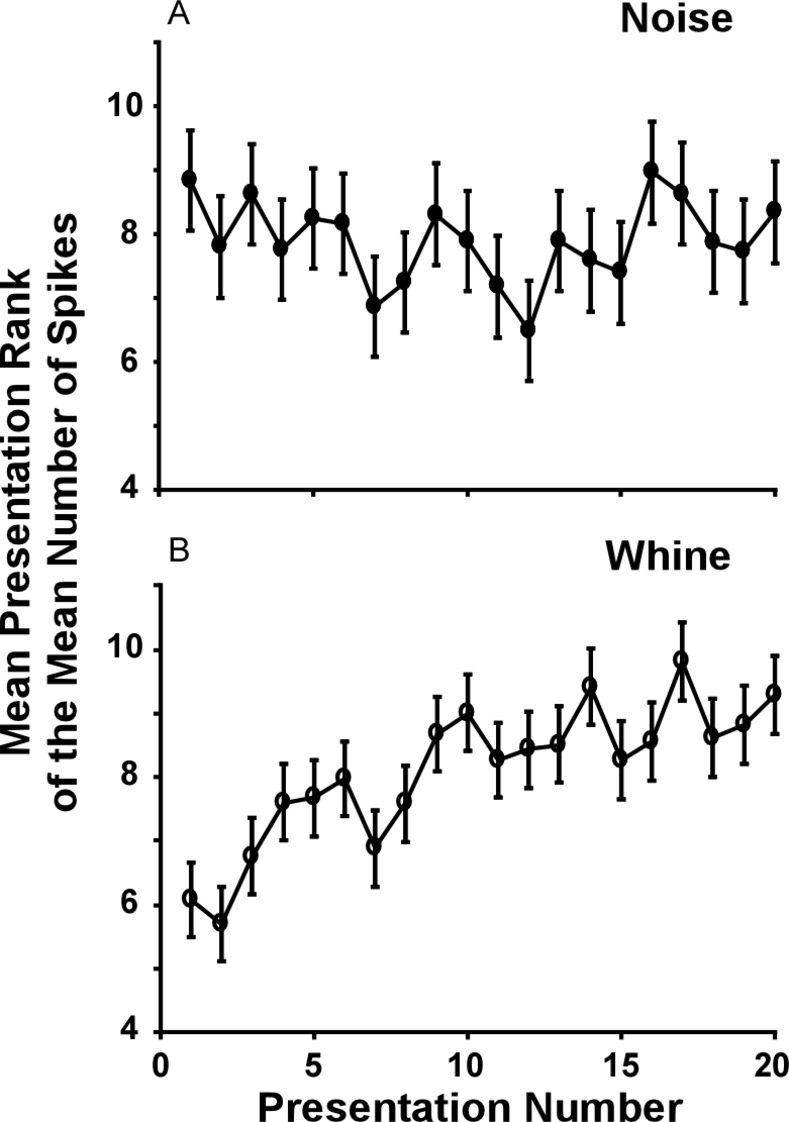
Assessing the potential contribution of adaption to greater whine than noise response. Mean response rank (N = 45 cells) for each of the stimulus presentations. There is evidence for adaption to the whine, but not to noise. Thus, adaptation in this experimental design cannot explain increased excitation to the whine relative to the noise. Note that because the number of action potentials per stimulus was often consistent, response rank included ties, compressing the range of ranks on the y-axis.

It is possible that the increased response (including the ‘bump’) to the whine over noise is due to greater adaptation to noise across the 20 stimulus presentations. Because of frequency specific adaptation [50], noise stimuli may be expected to elicit greater adaptation: noise continuously stimulates all critical bands, whereas stimulation of a critical band by the whine is limited to the time when modulated frequencies pass through them. Although adaptation was potentially present in the design of the previous behavioral experiments, too, and thus incorporated in the phonotactic responses [30], measurements here at the neural level enable analysis of such an effect. Within individuals that received all stimuli, each stimulus presentation was ranked by spike count, enabling analysis of the effect of presentation number. There was significant adaptation to the whine (earlier presentations had more spikes; p< 0.005), but not for the noise (p> 0.434; Fig 12): rank of stimulus presentation number (i.e., 1 to 20) decreased only for the whine. This is the *opposite* of that predicted if adaptation were to explain our finding of greater responses to the whine than noise responses. Thus, greater sensitivity to the whine does not appear to be due to a relative lack of adaptation.

## Discussion

Acoustic sexual signals exhibit inter- and intraspecific variance in multiple physical dimensions (e.g., time, amplitude, and frequency), enabling recognition and choice behavior [2, 74, 75]. Whereas measuring signal variance is often straightforward, measurements of a signal’s information-bearing elements and how the information-bearing parameters in those elements are processed by receivers are often difficult. The robust phonotaxis behavior in *P*. *pustulosus* frogs previously enabled isolation of the signal parameters mediating recognition behavior [30]. As described above, those behavioral data framed our tests of whether individual units in the *P*. *pustulosus* auditory midbrain match behavioral sensitivity and if those units, when summed in an experimental population, could predict behavioral discrimination of the call from noise.

### Neural correlates to behavior

Prior to the data in this study, neuronal mechanisms of call processing and discrimination in *P*. *pustulosus* frogs were assayed at a gross level. In response to long term exposure to stimuli (i.e., minutes), measurements of immediate early gene expression indirectly determined whether the amount of activity across many cells in an anatomical nucleus varied with stimulus type [54, 76–78]. These experiments guided our recordings, as they revealed a medial-to-lateral area of the torus that exhibits increased gene expressions for conspecific over heterospecific whines and other stimulus variants [68, 76]. However, even though IEG expression is correlated to excitation, the gene expression data cannot determine within-cell changes in responses, only that levels of expression across cells (and individual frogs) are increased for one stimulus and not for the other. The isolated recordings in our study overcame this limitation and further addressed whether changes in activity in individual cells were generated at a particular point in the stimulus, revealing a neural correlate to phonotaxis. Responses were significantly more sensitive to the whine, with closest responses to whine-like FM steps. Based on recording position, our sample of whine sensitive cells matches well the positions of cells exhibiting gene expression change in response to conspecific calls, as the medial-to-lateral area here overlaps the area of call-sensitive cells composing the laminar nucleus and dorsal area of the principle nucleus of the torus [68, 76]. This match offers independent confirmation of the call-sensitive area and suggests that the previously found call-induced change in IEG expression could be explained by within-cell changes in activity.

Does this bias towards whine sensitivity in these cells predict phonotactic decisions? Overall stimulus responses (Table 1) are grossly indicative of behavioral responses, including that the only response not significantly different from the whine’s was that to the 700–550 Hz FM step. However, aspects of the overall response do not align with behavior. For example, some of the largest overall discrimination values (d_a_, Table 1) should have been found for whine versus reverse whine, as the reverse whine does not elicit phonotaxis [30]. Instead, due to the large variance in these particular responses, this comparison had a small d_a_, smaller even than that for the whine vs. downward FM steps. Because comparing overall stimulus responses (Table 1) may include coding of non information-bearing parameters, we explored whether subsets of the response were indicative of behavior. Pooling the recordings across cells can address this question by creating ongoing response distributions that enable analysis of discriminability at the stimulus times predicted to be important for behavior. That is, the analysis asks in what part of the stimulus is discriminability highest. Experiments using phonotactic responses showed that the high-to-low frequency transition required for whine recognition occurs across an 80 Hz wide boundary: between 640–560 Hz [30]. For example, FM step stimuli, such as 700–550 Hz and 800–500 Hz, elicit significant whine-like behavioral choices when competing with noise. Consequently Wilczynski et al. [30] concluded, “stimulation anywhere between 900 and 560 Hz, followed at least 50 ms later by stimulation between 640 and 500 Hz in a natural whine is necessary for call recognition, although there is no single frequency in either region that must be stimulated for recognition to occur.” Interestingly, for the highest resolution bin width (1 ms) the neural responses to whine verses noise showed peak discriminability (highest d_a_) when the FM whine fundamental was at 601 Hz, which is centered in the behaviorally important FM transition zone. Additionally, the experimental stimulus that fit best into the behaviorally predicted range, the 700 Hz to 550 Hz FM step, was the only stimulus in which overall neural excitability did not differ from that of the whine (Table 1). These results support the conclusion that this random sample of whine sensitive cells is processing the information-bearing parameter for call recognition and is not simply exhibiting a more general increased sensitivity to whines, such as more action potentials across the whole call. For example, d_a_ values for whole responses were smaller than those around the FM transition at 600 Hz (see Table 1). This FM stimulus similarity between best neural discrimination and best phonotactic responses is somewhat tempered, however, by the value of the neural discrimination index, which at best was lower than that predicted from behavior. Here, for 1 ms bins d_a_ = 0.72, predicting 69–70% [79] whine versus noise responses. However, *P*. *pustulosus* females exhibit 100% (confidence interval to 83%) whine responses vs. noise [30], which would correspond to a much larger discrimination index (d_a_ > 3). This difference allows us to raise several caveats regarding whether these cells could mediate whine recognition as part of the sensory-motor interface of the midbrain [18, 39]. First, the sample population is undoubtably incomplete, likely missing cells that could have different response types as part of the recognition circuit. Second, the sample population could have included cells not involved in behavior, causing increased variance and/or reduced response difference. Both effects would decrease d_a_. Currently, however, there is no way to identify such cells for exclusion. Third, if the sample population is small compared to the endogenous circuitry, sampling error could affect variance, reducing discrimination. Indeed, larger neural sample sizes can increase neural discrimination towards that found in behavior [80]. Using the means (effect size) and variances of the 1 ms bin with the highest d_a_, we estimate that a sample size of >70 cells would be needed [81] to achieve the discrimination rates in behavior (>95%). The sample size here was 45. Fourth, there may be variance in sensitivity when frogs are under different endocrine states [82–84]. Although it is unlikely that females in the study were reproductive (none were gravid or had recently dropped eggs), male status was unknown. Reproductive modulation could increase sensitivity and potentially cause sexually dimorphic responses, although our data did not show this. Finally, these cells are recorded sequentially and across individuals. Thus, we cannot directly confirm how representative the recorded sample is using this ‘one-cell-at-a-time’ approach. However, given that the summed tuning curve from our sample population is nearly identical to those measured for responses of the entire torus and brainstem (Fig 3A [69, 85]), it appears the search technique did sample all auditory sensitivity in the frequency domain. Furthermore, the anatomy covered in our search area attempted to match the location of known midbrain auditory processing hubs [68, 76, 86]. When the tuning and recording locations are taken together with the sensitivity to the important FM transition, it suggests that a significant portion of the auditory midbrain exhibits specialized FM processing for the information-bearing parameter, as there was no evidence for generalized or varied FM sensitivity.

Midbrain sensitivity that is specialized to call features has been found across anuran taxa, including sensitivity to call spectrum [47, 48, 87]; amplitude modulation (AM) rates [40]; and sensitivity to interpulse intervals, enabling counting of call pulses [52, 88–90]. These specializations are correlated to behavior, likely contributing to signal recognition and discrimination decisions. Wilczynski and Ryan [17] noted that in spite of these specializations, responses of midbrain auditory neurons are not exclusive to calls (or other signals), as they often exhibit some, albeit less, response to many other stimuli and thus cannot explain phonotactic decisions that are exclusive to particular stimuli. Thus, such decisions were hypothesized to potentially be mediated by neurons at later processing nodes with greater integration and filtering (e.g., the thalamus; [91, 92]) and/or by the output of a network of cells (e.g., population response across anatomical nuclei). It is still unknown in *P*. *pustulosus* if cells with near categorical responses exist upstream, such as in the thalamus. However, sensitivity to the whine’s FM transition found here is arguably already that strong. Accounting for individual cell variance, comparisons showed significantly greater responses to the whine’s FM (the information-bearing parameter, in particular), suggesting extensive integration prior to the di- and telencephalon stages. This within-cell specialization raises the question of whether a standard model for population coding applies to whine processing in the túngara midbrain. Such models often include a population of neurons with different peak, yet overlapping, sensitivities across a range of a stimulus parameter, such as different FM. Because each cell’s sensitivity range is overlapping in such a model of population coding, a stimulus at one point on that range creates a distribution of responses across many cells. That response distribution across the population (i.e., its vector) creates the code for that stimulus [93]. *A priori*, we considered such a population vector as one possible outcome from our recordings: cells would show a range of FM sensitivity, including to different stimuli and even different parts of the whine. This would potentially create a population vector for whine stimuli. Hoke et al. [54], using *egr-1* induction, found that expression patterns across several midbrain nuclei were more effective in distinguishing acoustic stimuli than expression patterns in any one nucleus, which is consistent with a population code yielding a stimulus specific vector. However, we found little variance in cell sensitivity: the whine elicited the largest response across cells, suggesting that tuning in the *P*. *pustulosus* midbrain is strongly biased to whine FM and not a variety of FM types; the latter would be expected with population vector coding. Thus, in this sample of cells, there appears to be only one dominant sensitivity vector: one for the functional signal. More conclusive evidence for a lack of population coding would require more direct testing, however, such as using multi-unit recordings within individuals, which we did not do.

The FM tuning previously revealed through behavior and its neural correlate here appear most suitable for whine recognition behavior and less so for intraspecific call discrimination, as between-male variance in whine FM is small compared to the parameters of FM sensitivity. Ryan and Rand [61] analyzed over 300 calls from 50 males recorded in the field. Of 15 spectral and temporal call parameters, whine spectral components have relatively low coefficients of variation. And, the distributions of the initial and end frequencies of the whine’s FM sweep are on either sides of the important FM transition boundary. Thus, there appears to be little evidence that conspecific calls in the wild would not meet the criteria for FM sensitivity found here and in behavior [30].

### FM processing

Considering whine processing in a larger comparative context, FM sensitivity, including to functionally important sounds, is well known at the level of the vertebrate midbrain [37, 45, 94]. For mammals, FM sensitivity in the IC has been found in rodents [34, 43], but most data come from bats. Many units in the IC of bats are tuned to FM stimuli, with sensitivity varying with spectral and temporal structure, and mediated by multiple mechanisms residing potentially within and prior to the IC [35, 95–97]. Although many species use FM sounds, there are fewer data for non-mammalians. Comparatively, when added to those in mammals, our data and those in birds [41] strongly support the hypothesis that across-channel or integrative processing in the auditory midbrain is potentially expressed throughout vertebrate systems. Indeed, FM sensitivity in *P*. *pustulosus* expands understanding of spectral integration by units of frogs’ TS, including sensitivity to disparate frequency combinations important to call processing [87]. While it is still unknown if the mechanisms generating FM sensitivity in túngara frogs are the same as those in other taxa, responses here are consistent with varied temporal excitation and inhibition for different frequency channels, for example. GABAergic inhibition does shape the relationship between excitatory and inhibitory frequency tuning in the frog TS [98]. However, direct experimentation on whine sensitive units is needed to assess FM mechanisms. We would predict at least two benefits to such research. First, from a comparative point of view, it would address whether there are common solutions to creating FM sensitivity across disparate taxa. Second, unlike intraspecific whine FM variance, across species of túngara frogs there is prominent interspecific variance in the FM parameters of the whine-like component of the male calls, to which females can discriminate: conspecific versus heterospecific [99]. If FM sensitivity is mediated by asymmetric excitation and inhibition for frequency input, species-specific divergence in sensitivity could be mediated by shifting the integration across frequency channels [100].

## Conclusion

This study builds on extensive behavioral and gene expression assays of call sensitivity in *P*. *pustulosus*. Using isolated recordings of single cells in awake frogs, we found specialized sensitivity for the whine component of the complex male call. Functionally, this specialization was significant, as it matched what Suga [6] termed the information-bearing parameter of this species’ FM male call. Because each cell’s responses were dominated by this parameter (i.e., statistically significant excitation compared to other stimuli), our data suggest that population coding of the whine may not be necessary for stimulus recognition. However, based on signal detection theory analysis, we speculate that given such sensitivity by single cells, combining responses across cells would improve discrimination.
